# Proteome and Network Analysis Provides Novel Insights Into Developing and Established Chemotherapy-Induced Peripheral Neuropathy

**DOI:** 10.3389/fphar.2022.818690

**Published:** 2022-02-18

**Authors:** Larissa de Clauser, Christin Kappert, Julia R. Sondermann, David Gomez-Varela, Sarah J. L. Flatters, Manuela Schmidt

**Affiliations:** ^1^ Wolfson Centre for Age-Related Diseases, Institute of Psychiatry, Psychology and Neuroscience, King’s College London, London, United Kingdom; ^2^ Institute for Biomedicine, Eurac Research, Affiliated Institute of the University of Lübeck, Bolzano, Italy; ^3^ Max Planck Institute of Experimental Medicine, Goettingen, Germany; ^4^ Division of Pharmacology and Toxicology, Department of Pharmaceutical Sciences, University of Vienna, Vienna, Austria

**Keywords:** paclitaxel, neuropathy, proteomics, protein networks, dorsal root ganglia, chemotherapy, pain

## Abstract

Chemotherapy-induced peripheral neuropathy (CIPN) is a debilitating side-effect of cancer therapies. So far, the development of CIPN cannot be prevented, neither can established CIPN be reverted, often leading to the cessation of necessary chemotherapy. Thus, there is an urgent need to explore the mechanistic basis of CIPN to facilitate its treatment. Here we used an integrated approach of quantitative proteome profiling and network analysis in a clinically relevant rat model of paclitaxel-induced peripheral neuropathy. We analysed lumbar rat DRG at two critical time points: (1) day 7, right after cessation of paclitaxel treatment, but prior to neuropathy development (pre-CIPN); (2) 4 weeks after paclitaxel initiation, when neuropathy has developed (peak-CIPN). In this way we identified a differential protein signature, which shows how changes in the proteome correlate with the development and maintenance of CIPN, respectively. Extensive biological pathway and network analysis reveals that, at pre-CIPN, regulated proteins are prominently implicated in mitochondrial (dys)function, immune signalling, neuronal damage/regeneration, and neuronal transcription. Orthogonal validation in an independent rat cohort confirmed the increase of β-catenin (CTNNB1) at pre-CIPN. More importantly, detailed analysis of protein networks associated with β-catenin highlights translationally relevant and potentially druggable targets. Overall, this study demonstrates the enormous value of combining animal behaviour with proteome and network analysis to provide unprecedented insights into the molecular basis of CIPN. In line with emerging approaches of network medicine our results highlight new avenues for developing improved therapeutic options aimed at preventing and treating CIPN.

## Introduction

Chemotherapy-induced peripheral neuropathy (CIPN) is the major dose-limiting side-effect of several widely-used chemotherapeutics, affecting up to 70% of patients following standard chemotherapy regimens (Flatters et al., 2017; Seretny et al., 2014). Paclitaxel is a first-line treatment for ovarian and breast cancer. Hallmark symptoms of paclitaxel-induced neuropathy are painful hypersensitivity to mechanical and cold stimuli and sensory deficit in hands and feet. In patients, these symptoms cause significant difficulties in executing essential functions, such as pain while walking, inability to remove items from a fridge/freezer or difficulty in buttoning clothing (Flatters et al., 2017). The symptoms often persist for months, even years after chemotherapy, markedly impacting on quality of life (Mols et al., 2014; van den Bent et al., 1997). Thus, patients may well be cancer-free, but suffering a debilitating painful neuropathy as a result of their cancer treatment (Flatters et al., 2017). There is no effective pharmacological therapy for the prevention or treatment of CIPN with many established analgesics shown to be ineffective [reviewed in (Hershman et al., 2014)]. Thus, the emergence of neuropathy causes dose reduction or cessation of otherwise effective cancer treatment, potentially limiting patient survival. Greater understanding of the molecular actors and pathways driving CIPN will enable the development of more effective therapies for paclitaxel-induced neuropathy. Additionally, the opioid crisis has highlighted the necessity for mechanism-based treatment options with higher efficacy and specificity.

Both genomics and transcriptomics are increasingly exploited in pain research to characterize global changes at multiple levels of the nociceptive system. In the case of CIPN, several studies have investigated changes in the transcriptome at the level of the dorsal root ganglia (DRG) in rodent models (Bangash et al., 2018; Megat et al., 2019; Housley et al., 2020; Kim et al., 2020). However, results at mRNA level correlate only up to 50% with factual levels of corresponding proteins, which renders the interpretation of results and identification of novel targets difficult (Liu et al., 2016; Reimegård et al., 2021). Hence, a thorough understanding of CIPN pathological dynamics warrants studies at the level of the proteome. The rat, in particular, constitutes a useful *in vivo* model of CIPN (Lehmann et al., 2019), as the human and rat DRG proteome are largely conserved (Schwaid et al., 2018). Here, we combined the established rat model of CIPN (Flatters and Bennett, 2006; Duggett et al., 2016; Duggett et al., 2017) with quantitative and comprehensive mass spectrometry-based proteomics to reveal proteome dynamics in rat DRG in an unbiased manner. Importantly, we did so at two pathologically relevant time points. Firstly, pre-CIPN, i.e., at day 7, corresponding to 1 day after the last injection of paclitaxel and prior to neuropathy development, evidenced by lack of apparent mechanical hypersensitivity. Secondly, peak-CIPN, i.e., around day 28, when neuropathy and paclitaxel-induced mechanical hypersensitivity are fully developed despite the fact that paclitaxel treatment finished at day 6 - a phenomenon, which is referred to as “coasting.” To enhance the translational significance of our work (i) we chose a CIPN model induced by a relatively low dose of paclitaxel (Flatters and Bennett, 2006; Duggett et al., 2016; Duggett et al., 2017; Griffiths et al., 2018); (ii) we mimicked clinically used cycles of chemotherapy by repetitive paclitaxel application; (iii) we prepared a clinically relevant formulation of paclitaxel (please see Methods for details); (iv) we present relevant symptoms of mechanical hypersensitivity as well as coasting; and (v) we performed all animal studies in a blinded and fully randomised manner. Our results identify changes in discrete proteins and cellular pathways providing unprecedented insights into protein network dynamics following paclitaxel treatment. Even more, we show how changes in the proteome are longitudinally altered in relation to the development and maintenance of paclitaxel-induced peripheral neuropathy. Thus, our data represent a highly valuable resource for further mechanistic studies and uncover novel potentially druggable targets for the prevention and treatment of CIPN.

## Experimental Procedures

### Experimental Design and Statistical Rationale

To determine global changes in DRG during the development and in established chemotherapy-induced peripheral neuropathy, 32 rats were injected with either paclitaxel or vehicle. After assessment for pain-related behaviours, DRG from two rats per condition (vehicle/paclitaxel) were pooled at pre-CIPN (24 h after the last paclitaxel/vehicle injection) and peak-CIPN (25–33 days after the last paclitaxel/vehicle injection), resulting in four biological replicates per condition and timepoint. Samples were further processed and analyzed by DIA-MS. Benjamini-Hochberg (BH)-adjusted p-values (= q-value) were used for multiple testing and significantly altered proteins were defined by setting a cutoff of q < 0.05.

### Animal Husbandry

Adult male Sprague-Dawley rats (175–200 g, Envigo) were housed in groups of three or four in a temperature-controlled room with a 12-h light/dark cycle (7a.m. lights on). All cages contained sawdust bedding with environmental enrichment materials and food/water was freely available. All procedures were conducted in strict accordance with the UK Animals (Scientific Procedures) Act, 1986, approved by King’s College London animal welfare committee under the UK Home Office project license PBA346803.

### Administration of Paclitaxel

Clinically formulated paclitaxel solution for infusion (6 mg/ml; Accord Pharmaceuticals UK) was diluted with 0.9% sterile saline (Fresenius Kabi, United Kingdom) to achieve a 2 mg/ml solution for injection. To replicate the clinical formulation, a vehicle stock solution was made using 1:1 solution of cremophor EL (Sigma, United Kingdom) and ethanol plus 2 mg/ml sodium citrate (Sigma, United Kingdom). Prior to administration one part vehicle stock solution was diluted with two parts 0.9% sterile saline. Rats received an intraperitoneal injection of 2 mg/kg paclitaxel or equivalent volume of vehicle solution, on four alternate days (0, 2, 4, 6) as previously described (Duggett et al., 2017; Griffiths et al., 2018). This dosing paradigm did not cause weight loss or any other impairment to animal health.

### Assessment of Chemotherapy-Induced Neuropathy

Rats were habituated to the testing environment for ∼20 min on two or three separate occasions before baseline testing. Mechanical hypersensitivity was assessed using von Frey filaments (Touch-Test^TM^ Sensory Evaluators, Linton Instrumentation, United Kingdom) as previously described (Griffiths et al., 2018). Rats were placed in elevated, clear Perspex boxes (15 cm × 16 cm x 21 cm) with a wire-rung floor and allowed to acclimatise. After habituation, three baseline measurements were taken on separate days prior to administration of paclitaxel/vehicle. To measure mechanical hypersensitivity, when still alert and with all four paws in contact with the floor, von Frey filaments (4, 8, 15 g) were applied to the hind paws of each animal, in ascending order of force. Each von Frey filament was applied for 5 s to five different points on the mid-plantar region of one hind paw, after moving to the next hind paw. The number of withdrawals from each hind paw were added together to give a withdrawal score out of ten for each von Frey filament. Two critical time points of paclitaxel-induced peripheral neuropathy were investigated in this study: pre-CIPN, i.e., day 7 corresponding to 24 h after the last injection of paclitaxel and peak-CIPN, ∼day 28 after the first injection of paclitaxel. These phenotypic timepoints of the paclitaxel rat model are well-established and previously reported (Fidanboylu et al., 2011; Griffiths and Flatters, 2015; Duggett et al., 2016; Duggett et al., 2017; Griffiths et al., 2018). Some animals from the peak-CIPN group were also tested for mechanical hypersensitivity at day 7 and these data were included in the behavioural analysis at pre-CIPN.

### Blinding and Randomisation

All behavioural testing was performed by the same experimenter and in a blinded manner, to avoid unconscious bias. Following baseline testing, animals were split into two equal groups based on their baseline response to von Frey stimulation. Paclitaxel/vehicle treatments were randomised within each cohort of animals. Behavioural extraneous variables were minimised by testing concurrent vehicle-treated groups of equal size throughout all experiments.

### Western Blot

Lumbar DRG were dissected from individual rats (vehicle: *n* = 6 rats, pre-CIPN: *n* = 5 rats) following standard procedures, in brief: rats were sacrificed, the dorsal part of the rib cage and the spinal column were removed, and the spinal column was carefully cut open at the dorsal side. The spinal cord was carefully pushed to the side and lumbar DRG (between L6–L3 spinal levels, i.e., 6 DRG/rat) were pulled out from their grooves using fine forceps. DRG were immediately frozen in liquid nitrogen until further use. Total protein was obtained by homogenization in RIPA lysis buffer containing protease and phosphatase inhibitors. 30 µg samples were separated on a 10% polyacrylamide gel by SDS-PAGE and transferred to PDVF membrane (0.45 µm, Millipore). Blots were blocked and incubated overnight at 4°C in rabbit anti – β-catenin (1:500, ab32572, Abcam, United Kingdom) and mouse anti –β-actin (1:5000, A5441, Merck, United Kingdom). The following day the membrane was incubated for 1 h at room temperature in goat anti-mouse IRDye® 680 (1:5000, 926-68070, LI-COR) and goat anti-rabbit Dylight 800 (1:5000, A23920-1, Abbkine) and scanned with the Odyssey Infrared Imaging System (LI-COR Biosciences). Densiometric analysis was performed using ImageJ (version 1.52p) and by normalizing expression to β-actin.

### Protein Isolation for DIA-MS

At indicated time points six lumbar DRG were extracted per rat between L6–L3 spinal levels (for dissection procedure please see section above), placed into a plastic microtube, flash frozen in liquid nitrogen and stored at −80°C until shipment on dry ice. DRGs from 8 rats/condition were pooled in four biological replicates each (i.e., DRG from 2 rats/replicate). Protein isolation was performed as described previously (Bruderer et al., 2017). In brief, the frozen tissue was homogenized in 4% SDS lysis buffer (4% SDS in 100 mM Tris, 10 mM DTT, 5% glycerol, complete protease inhibitor cocktail (Roche), pH 7.5). Following, the homogenate was incubated at 70°C for 10 min. The homogenate was centrifuged at 10,000 × g for 5 min at room temperature to remove cell debris. Proteins were precipitated by the addition of 5 × volume pre-chilled acetone (Roth) and incubated for 2 h at −20°C. The protein precipitate was centrifuged at 14,000 × g for 30 min, the pellet washed with ice-cold 80% ethanol (AppliedChem), and centrifuged again at 14,000 × g for 30 min. The proteins were air-dried and resuspended in 2% SDS lysis buffer.

### Sample Preparation for DIA-MS

Sample preparation followed the single-pot, solid-phase-enhanced sample preparation (SP3) protocol (Hughes et al., 2019). In short: precipitated proteins (in 2% SDS lysis buffer) were reduced at 60°C for 30 min at 1,000 rpm in a ThermoMixer. Samples were alkylated in the presence of 20 mM Iodoacetamide (IAA) in the dark at room temperature. The reaction was quenched with a final of 5 mM DTT for 15 min. Paramagnetic beads (BD) were prepared following the manufacturer’s instructions. Binding of proteins and beads was facilitated by the addition of ethanol in a 1:1 ratio (vol/vol) for 5 min at 24°C and 1,000 rpm agitation. Detergent removal was achieved by three off-magnet rinses with 80% ethanol. After complete removal of washing solution, Trypsin/Lys-C (Promega, Madison, WI, United States) was added in a 1:25 ratio (enzyme: protein) in 100 mM ammonium bicarbonate (pH 8). Proteolysis was performed overnight at 37°C in a ThermoMixer at 1,000 rpm to prevent aggregation of beads. After 16 h, elution of proteolytic peptides was accomplished by placing the Eppendorf tube on a magnetic rack and transfer of the supernatant to a fresh tube. Tryptic peptides were acidified to a final concentration of 0.5% trifluoro-acetic acid (TFA) and desalted with commercially available C18 columns (UltraMicroSpin, The Nest Group). The clean-up protocol followed the manufacturer’s instructions. Desalted peptides were dried in a centrifugal evaporator and stored at −80°C.

### Data Independent Acquisition Mass Spectrometry

Samples were analyzed on a Q-Exactive HF-X Orbitrap mass spectrometer coupled to an UltiMate 3000 UHPLC System (both Thermo Fisher Scientific, Bremen, Germany). 250 ng of tryptic peptides were loaded and trapped on an Acclaim^TM^ PepMap^TM^ column (2 cm; 5 µm C18 packing material; Thermo Fisher Scientific) at a flow rate of 8 μL/min with 0.1% (v/v) TFA for 10 min. Washed analytes were separated on a 75 µm × 25 cm Acclaim^TM^ PepMap^TM^ column (2 µm C18 packing material; Thermo Fisher Scientific) at a flow rate of 300 nL/min using buffer A [1% (v/v) ACN, 0.1% (v/v) FA] and buffer B [85% (v/v) ACN, 0.1% (v/v) FA]. Peptides eluted along a linear gradient (22%–55% buffer B, 90 min) to the mass spectrometer *via* a nano-electrospray ion source (Thermo Fisher) followed by a 10 min wash with 90% buffer B. Column temperature was constantly kept at 60°C. MS raw data were analyzed in a positive ion mode using a data-independent acquisition mode (DIA). Each MS1 scan in the range of 350–1,204 m/z [automatic gain control target (AGC) value of 3e6 or 60 ms injection time] at a resolution of 120,000 FWHM was followed by MS2/DIA scans at 30,000 FWHM (AGC of 3e6, auto for injection time). Selected precursor ions were fragmented using higher-energy collision-induced dissociation (HCD) with stepped (25, 27, 30) normalized collision energy (NCE). Isolation windows were set to 14 m/z resulting in 61 spectra per DIA segment. MS1 and MS2 spectra were recorded in profile mode, the default charge state was two.

### DIA-MS Data Analysis

MS/MS raw files were analyzed using the Spectronaut software version 13.11.200127 (Biognosys, Switzerland). Spectra were searched against the rat Uniprot FASTA database (“accessed Oktober 2019”) and a common contaminants database by the Pulsar^TM^ search engine (Tsou et al., 2015) using two analysis pipelines: the spectral library-based search and the directDIA^TM^ workflow developed at Biognosys (Supplementary Table S1). Cysteine carbamidomethylation was defined as fixed modification while acetylation of protein N-term and methionine oxidation were set as variable modifications. Enzyme specificity was set to Trypsin in consideration of the proline effect, allowing two miscleavages within a minimum peptide length of 7, and a maximum peptide length of 52 amino acids. The false discovery rate (FDR) for precursor, protein, and peptide identification was set to 0.01 per run by searching a decoy database. Label free quantification was based on the Biognosys Factory settings by Spectronaut but excluding single peptide hits and considering the top 8 for quantity calculations. Quantitation was executed on MS2-level using the area under the curve and data were filtered by Qvalue sparse (precursors robustly found in at least one sample). For each biological condition the coefficient of variation (CV) among biological replicates was at approx. 20%. Statistical testing of differential protein abundances between conditions was calculated in Spectronaut for each protein ID by performing a pairwise t-test. Benjamini-Hochberg (BH)-adjusted p-values were used for multiple testing and significantly altered proteins were defined by setting a cutoff of q < 0.05. Significantly regulated proteins from both analysis pipelines were pooled in a combined candidate list and duplicates as well as single-peptide-hits removed (Supplementary Table S1). Any potential keratin and serum albumin contaminations were also removed. Volcano plots were generated in GraphPad 8.0 with data obtained from the spectral library-based pipeline complemented with the combined hit list (q < 0.05) of both analysis pipelines. All lists were compared based on unique gene names as often multiple protein entry IDs exist for a given protein candidate. Heatmaps and PCA plots (Supplementary Figure S1) were generated with the Perseus software platform (https://maxquant.net/perseus/) upon upload of combined raw data as aforementioned for volcano plots. All raw data, sample report files, and plots visualizing the coefficient of variation for each analysis pipeline have been deposited to the ProteomeXchange Consortium via the PRIDE partner repository (Perez-Riverol et al., 2019) with the dataset identifier PXD028356.

### Gene Ontology Analysis

The list of significantly regulated proteins (BH-adjusted p-value, q-value < 0.05) was uploaded to the web interface STRING (https://www.string-db.org/) and QIAGEN Ingenuity Pathway Analysis (IPA) (Krämer et al., 2014). For STRING analysis, only significant enrichments (FDR <0.05) in cellular component biological process (BP) and REACTOME pathways are reported. STRING settings for network visualization: Confidence view; confidence level 0.7; clustering algorithm MCL set to 3. Shown results from IPA analysis correspond to diseases and function analysis, functional analysis of a network, and canonical pathway analysis. The significance of the association between the experimental data set and IPA resources was measured by a right-tailed Fisher’s Exact Test (p-value). Note that this analysis is performed based on unique gene names of identified candidates, as these are more frequently used and recognized within the scientific community. Thus, for clarity, we additionally indicate gene names in the text wherever they differ from protein entries in the UniProt database (uniprot.org).

### Comparisons With Other Datasets

For comparison with other datasets only proteins with an annotated gene were considered (289/295) (see also Supplementary Table S2). To determine if the identified proteins under basal conditions are expressed within a particular subset of sensory neurons, we compared gene names to single cell RNAseq data from the mouse (Zeisel et al., 2018). A particular gene and corresponding protein were defined part of a subset if at least 25% of cells within that subset expressed the transcript. Proteins were defined as neuronal if they were expressed in at least one sensory neuron subset. For comparisons with other CIPN transcriptome datasets, criteria were defined as indicated in Supplementary Table S7. For microarray datasets (Bangash et al., 2018; Housley et al., 2020) the data was downloaded and analyzed with Transcriptome Analysis Console (TAC Ver 4.0, Thermo Fisher). For RNAseq data (Kim et al., 2020) and translating ribosome affinity purification (TRAP)-processed data (Megat et al., 2019) was directly accessed through the publication. Venn diagrams for 2–3 groups were generated with BioVenn (Hulsen et al., 2008), while those for four groups with InteractiveVenn (Heberle et al., 2015).

### Statistical Analysis

Sample sizes were not determined a priori, yet they are in line with previous studies in the field. All replicates were biological. Details on statistical analysis for each experiment are given in the respective figure legends and methods section. GraphPad 8.0 was used for statistical analysis of behavioural and western blot data as well as for the visualization of data in volcano plots. Data is represented as mean ± SEM, *p < 0.05, **p < 0.01.

## Results

### Rat Behaviour Combined With Unbiased Quantitative Proteomics Reveals Proteome Dynamics Prior to and During Paclitaxel-Induced Neuropathy

**FIGURE 1:**
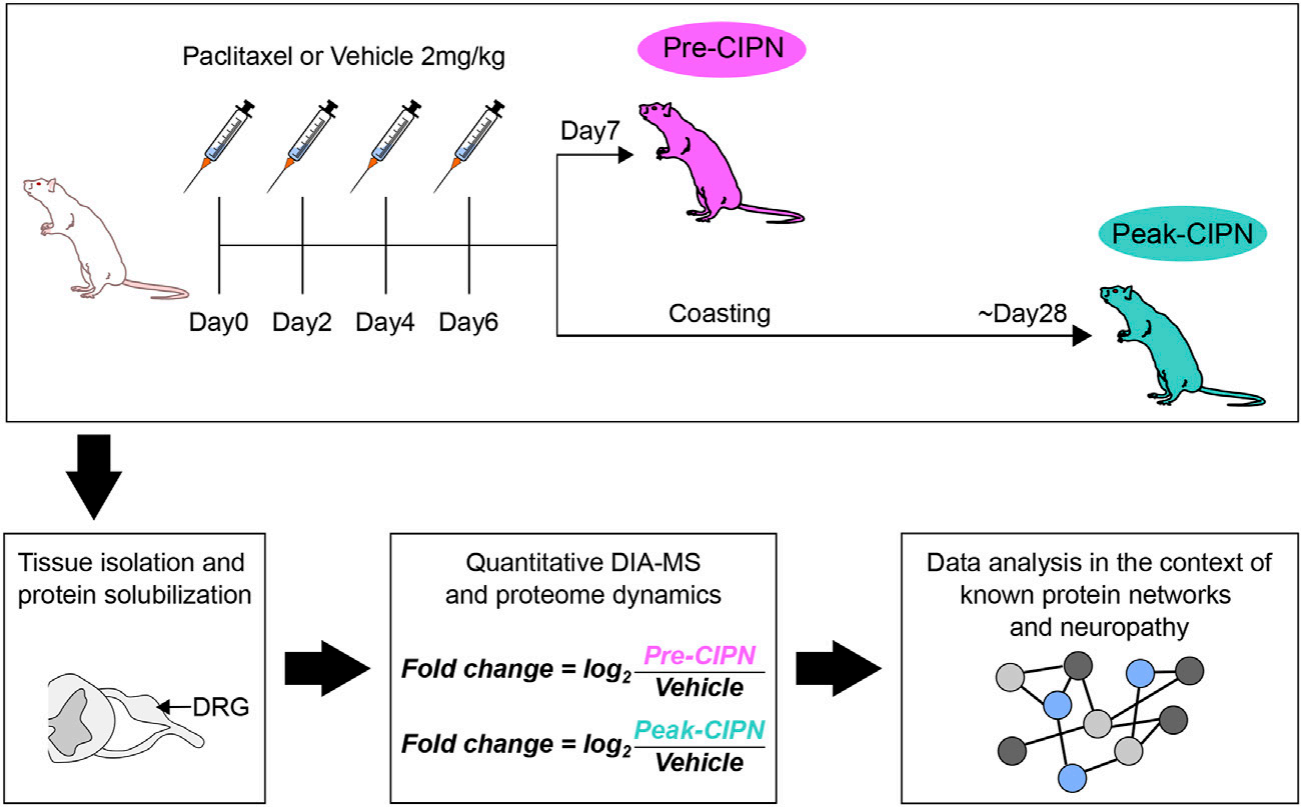
Schematic overview of the integrated workflow: from paclitaxel or vehicle administration *via* behavioural assessment of neuropathy, protein solubilization, and quantitative DIA-MS to analysis of altered protein networks.

**FIGURE 2:**
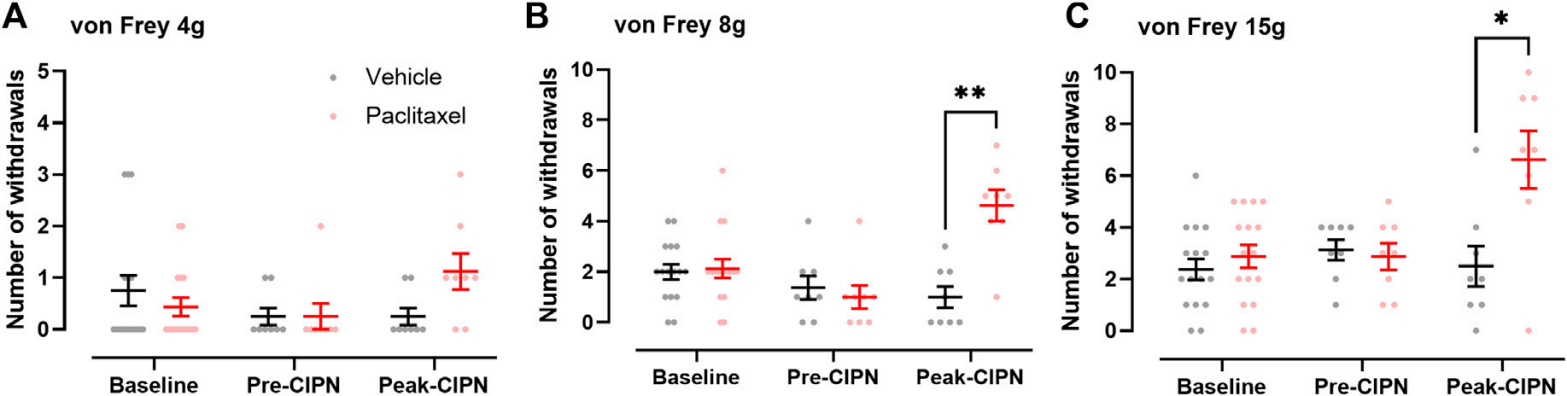
Time course of paclitaxel-induced mechanical hypersensitivity. **(A–C)** Number of withdrawals (/10) in response to stimulation of the hindpaw with von Frey filaments of 4 g **(A)**, 8 g **(B)** and 15 g **(C)** at baseline (*n* = 16 per group), pre-CIPN (day 7; vehicle *n* = 13, paclitaxel *n* = 14) and peak-CIPN (day 25–33; *n* = 8 per group). Individual values and mean ± SEM for vehicle (black) and paclitaxel (red) are shown. Mixed-effects model with Holm Sidak correction (*p < 0.05, **p < 0.01). Note that in **(A)**, a different axis scale was chosen to better visualize paclitaxel-induced changes. Note that some animals from the peak-CIPN group were also tested for mechanical hypersensitivity at day 7 and these data were included in the behavioural analysis at pre-CIPN accounting for the differences in sample sizes.

We used an integrated workflow from rat behaviour *via* quantitative proteomics to network analysis to comprehensively study proteome dynamics upon paclitaxel treatment in rat DRG (Figure 1). To determine the development of paclitaxel-induced neuropathy, we measured mechanical hypersensitivity to 4, 8 and 15 g von Frey filaments before, at day 7 and at ∼ day 28 following paclitaxel/vehicle administration. Consistent with our previous work (Duggett et al., 2016; Duggett et al., 2017; Griffiths et al., 2018), we found that rats gradually develop mechanical hypersensitivity in response to four low systemic doses of paclitaxel, administered on days 0, 2, 4, and 6. As expected, tactile hypersensitivity in response to both 8 and 15 g von Frey filaments peaked at around 4 weeks after paclitaxel treatment (Figure 2). Two separate cohorts were generated for pre-CIPN and peak-CIPN DRGs used in this study.

### Differential Regulation of Proteins by Paclitaxel is More Prominent Prior to Neuropathy Development

**FIGURE 3:**
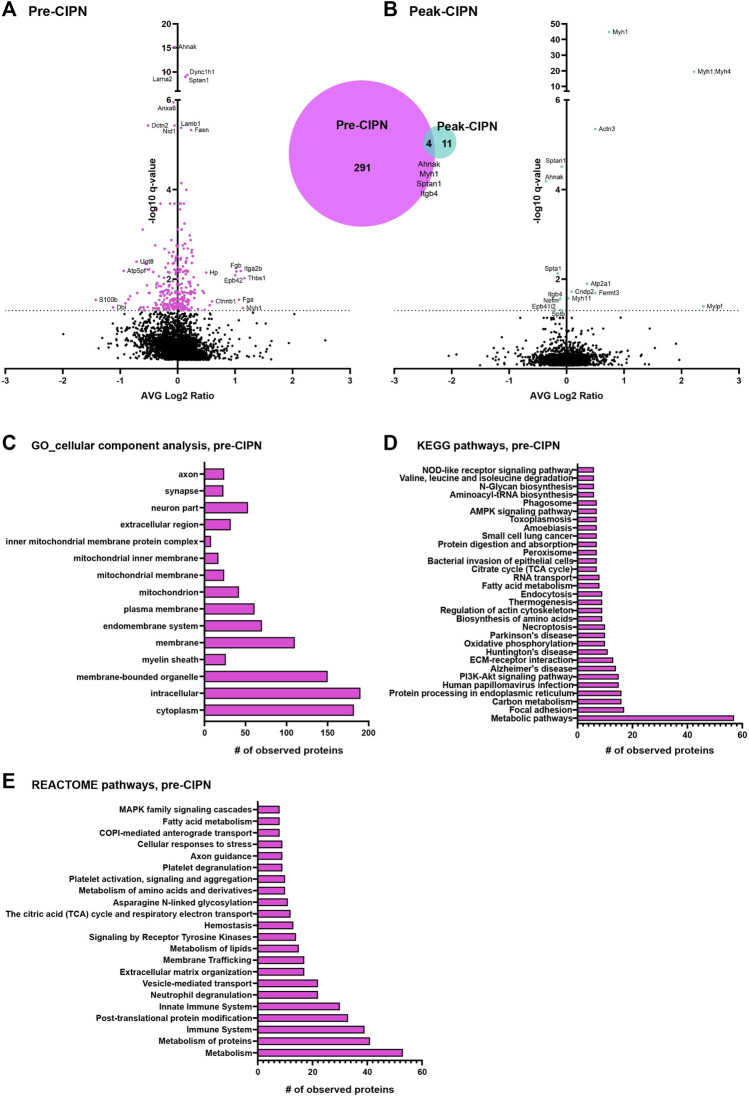
DRG protein signature following paclitaxel treatment prior to (pre-CIPN) and during (peak-CIPN) neuropathy. **(A)** The Volcano plot displays the average log2 fold change (log2 pre-CIPN/vehicle) of detected proteins and corresponding -log10 of q-values. 295 out of a total of 3,228 quantified proteins were significantly regulated (magenta-colored circles above the dotted horizontal line, which represents q < 0.05; q = BH-adjusted p-value) following paclitaxel treatment at pre-CIPN time point compared to vehicle treatment. The numbers are summarized in the Venn diagram. Examples of regulated proteins are given, yet, for clarity and consistency within the manuscript, gene names are used. **(B)** The volcano plot displays the average log2 fold change (log2 peak-CIPN/vehicle) of detected proteins and corresponding -log10 of q-values. 15 out of a total of 3,224 quantified proteins were significantly regulated (cyan-colored circles above the dotted horizontal line, which represents q < 0.05; q = BH-adjusted p-value) following paclitaxel treatment at peak-CIPN time point compared to vehicle treatment. **(C)** At pre-CIPN regulated proteins are localized to diverse cellular components as assessed by gene ontology (GO) analysis. **(D, E)** At pre-CIPN, regulated proteins are associated with diverse KEGG **(D)** and REACTOME **(E)** pathways. Only annotated pathways, which exhibit significant enrichment (FDR <0.05; assessed *via* the web-based interface STRING); are reported.

To determine changes at the proteome level we used our established proteomics-workflow, i.e., liquid chromatography coupled mass spectrometry (LC-MS) in data-independent acquisition mode (DIA). We quantified >3,200 protein groups (please see methods for further details and identification criteria) at pre-CIPN and at peak-CIPN across all biological replicates (Figures 3A,B; Supplementary Table S1). While biological replicates of each condition exhibited a certain degree of variability with limited co-clustering (Supplementary Figure S1), PCA analysis showed the expected separation of experimental conditions at peak-CIPN (Supplementary Figure S1B). Upon statistical comparison (corrected for multiple testing using the Benjamini-Hochberg method, q-value (Storey, 2002); for complete dataset, please see Supplementary Table S1), we observed little overall proteome changes as visualized in the volcano plot (Figures 3A,B). Specifically, 295 protein groups were significantly regulated at pre-CIPN (Figure 3A), while only 15 protein groups were altered at peak-CIPN (Figure 3B and Supplementary Table S2: quantification was performed with at least two identified precursors; please see methods for further details and identification criteria). Interestingly, four candidates (myosin-1, *Myh1*; AHNAK nucleoprotein, *Ahnak*; spectrin alpha chain, *Sptan1*; integrin beta, *Itgb4*) showed robust regulation at both time points.

### Paclitaxel Alters Multiple Biological Pathways in Rat DRG

We then used the web-based STRING interface (https://www.string-db.org/) with inbuilt gene ontology (GO) annotations, and QIAGEN Ingenuity Pathway Analysis (IPA) to visualize predicted protein networks and pathway annotations of regulated proteins at pre-CIPN. Due to the minimal amount of regulated proteins at peak-CIPN we did not perform pathway analysis with the peak-CIPN dataset. Regulated proteins at pre-CIPN were associated with diverse cellular compartments including the plasma membrane and also the mitochondrion just to name a few (Figure 3C). Furthermore, 28 significantly regulated proteins at pre-CIPN are included in a previously published mitochondrial proteome dataset (Supplementary Figure S2A). Using IPA, mitochondrial dysfunction emerged with high significance in IPA Tox listing and is among the top regulated canonical pathways (Supplementary Table S3) Regulated proteins associated with mitochondrial dysfunction (13 proteins, for details please see Supplementary Table S3) can mainly be attributed to complexes I, II, and V (Supplementary Figure S2B). Indeed, mitochondrial dysfunction has been shown to be a major player in CIPN. Electron micrographs of patient biopsies show the presence of vacuolated mitochondria in sensory axons (Fazio et al., 1999; Ebenezer et al., 2014). The presence of mitochondria with atypical, swollen morphology has also been demonstrated in preclinical models of CIPN at the level of the saphenous nerve, sciatic nerve, DRG, and sensory axons in the dorsal root (Trecarichi and Flatters, 2019). In addition, in sensory neurons, the axonal transport of nuclear mRNA encoding for mitochondrial proteins implicated in mitochondrial fusion/fission appears to be disrupted and correlates with mitochondrial dysfunction (Bobylev et al., 2015). In line with these data, drugs targeting mitochondria have shown antinociceptive effects. ROS (reactive oxygen species) scavengers attenuate the development of CIPN in the rat (Kim et al., 2010; Fidanboylu et al., 2011) and inhibition of complex III has similar effects (Griffiths and Flatters, 2015). In essence, our data on paclitaxel-induced proteome alterations may help identify distinct future targets to prevent and/or re-align mitochondrial defects before CIPN has fully developed.

In accordance with IPA-based analysis, KEGG (Figure 3D) and Reactome (Figure 3E) pathway analysis suggested that, at pre-CIPN, regulated proteins were associated with major cellular pathways such as those related to the extracellular matrix (ECM), metabolism, mitochondrial processes, and endocytosis. These data may, in part, reflect acutely toxic effects of paclitaxel treatment (Reeves et al., 2012; Loprinzi et al., 2007). Interestingly, Reactome analysis suggested prominent alterations of the immune system including platelets as well as neutrophils (Figure 3E), and thus, provide unprecedented insights into distinct proteins potentially involved in these changes. Notably, chemotherapeutics not only exert systemic immune modulation, but can also result in neuroinflammatory responses at the level of the PNS (Lees et al., 2017). Several pro-inflammatory cytokines (TNF-α, IL-1β, and IL-6) are induced at the level of the DRG (Kim et al., 2019; Zhang et al., 2019) and spinal cord (Salvemini et al., 2012; Janes et al., 2015) in response to chemotherapy. Importantly, inhibition of TNF-α or IL-1 signaling in a rat model of paclitaxel-induced neuropathy was able to attenuate mechanical and cold hypersensitivity (Al-Mazidi et al., 2018). Similarly, mice lacking IL-6 or pre-treated with an IL-6-neutralizing antibody were protected from mechanical hypersensitivity induced by paclitaxel (Huehnchen et al., 2020). These inflammatory mediators can be released by a variety of cells, including satellite glial cells, Schwann cells, macrophages and neutrophils (Uhelski et al., 2021). Indeed, a single injection of paclitaxel is sufficient to recruit innate immune cells, including macrophages, monocytes, and neutrophils to the DRG (Liu et al., 2014). It remains to be investigated if the activation of these cells is important for the production of cytokines and if they contribute to hypersensitivity. In line with our data, an immune response signature was also identified in a recent transcriptome study of mice with CIPN, in particular in response to vincristine (Starobova et al., 2020), which, like paclitaxel, prevents mitosis by acting on microtubules (Jordan and Wilson, 2004).

### The Majority of Proteins Altered at Pre-CIPN Are Neuronal

**FIGURE 4:**
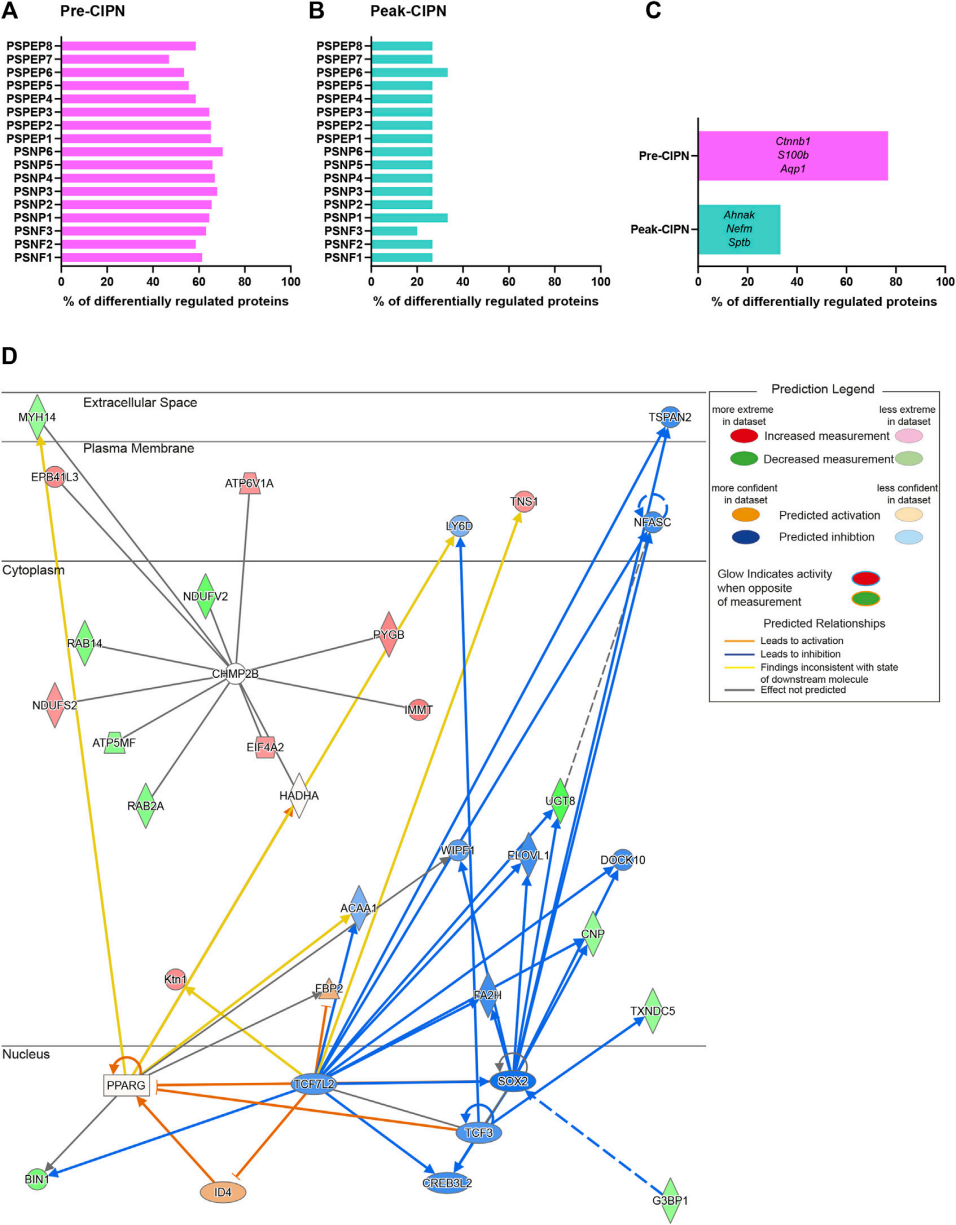
A major fraction of differentially regulated proteins at pre-CIPN is expressed in sensory neurons. **(A/B)** Percentage of differentially regulated proteins at pre-CIPN **(A)** and peak-CIPN **(B)**, for which the corresponding transcript is expressed in at least 25% of mouse sensory neurons within each subpopulation according to (Zeisel et al., 2018). **(C)** Percentage of differentially regulated proteins that are present in at least one of the neuronal subsets. Examples are given (using gene names). **(D)** Predicted activity analysis (by IPA) of a protein network associated with “neurological disease” (Supplementary Table S5, Network 7) harbouring regulated proteins at pre-CIPN.

**FIGURE 5:**
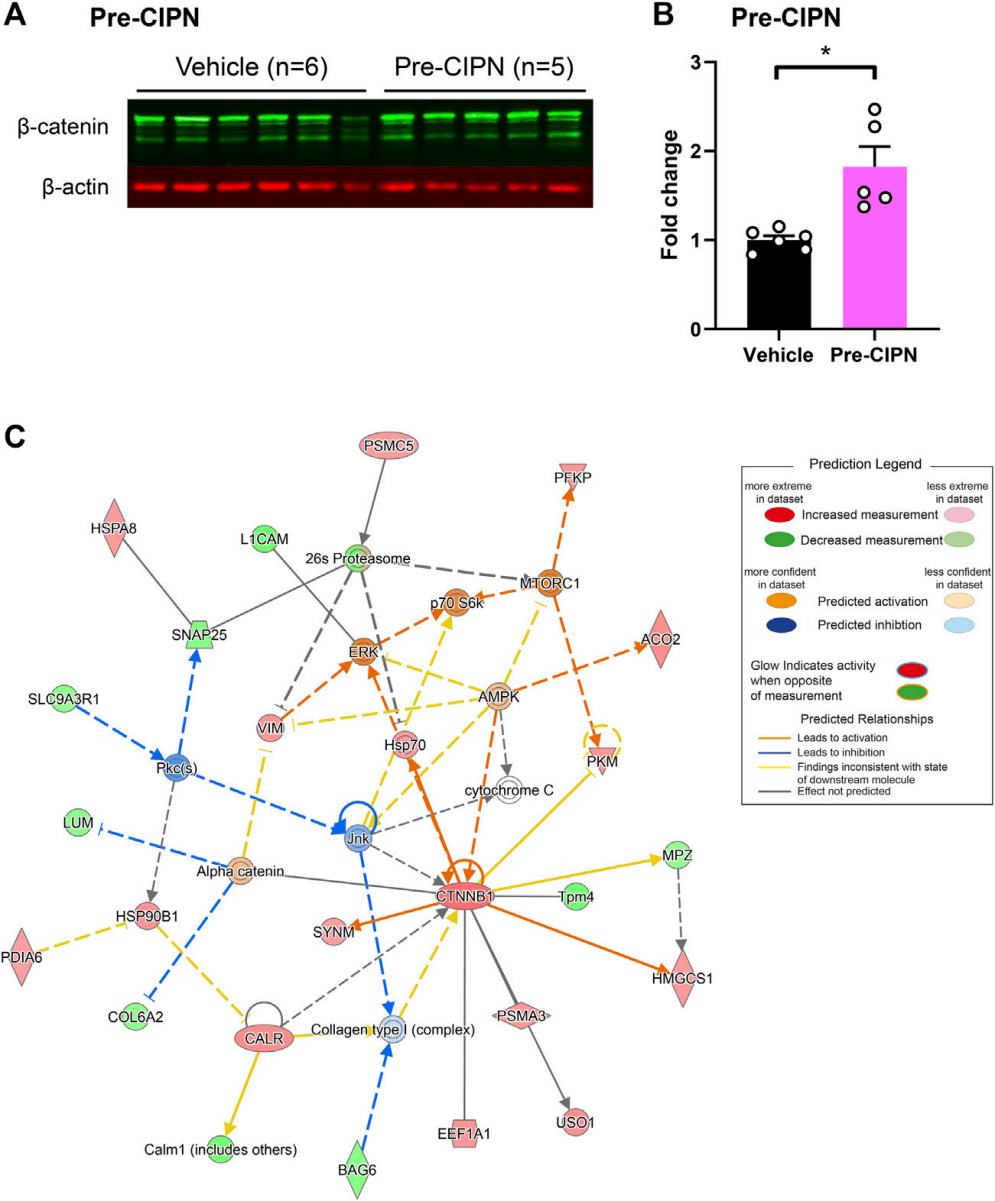
Upregulation of ß-catenin (CTNNB1) with pronounced effects on associated protein networks. **(A)** Western Blot for β-catenin (green bands) and β-actin (red bands) in vehicle (*n* = 6) and pre-CIPN (*n* = 5). **(B)** Quantification of band intensity normalized to β-actin and expressed as fold change relative to control. Two-tailed unpaired t-test; p-value: 0.0036. **(C)** Protein network associated with CTNNB1 (Supplementary Table S5 Network 5) and predicted activity analysis by IPA.

To determine the proportion of neuronal proteins within our dataset, we compared our candidate list to the corresponding transcripts from single-cell RNA sequencing data of mouse DRG neurons (Zeisel et al., 2018), as no rat data is currently available. The transcripts corresponding to 276/289 (95.52%) differentially regulated proteins (DRPs) at pre-CIPN, and 15/15 (100%) at peak-CIPN were present in the mouse dataset (Supplementary Table S4). DRPs at pre-CIPN (Figure 4A) and peak-CIPN (Figure 4B) did not show enrichment in a specific neuronal subset. Overall, 222/276 (80.43%) of DRPs at pre-CIPN and 5/15 (33.33%) at peak-CIPN were identified as neuronal (Figure 4C; Supplementary Table S4). The involvement of neuronal proteins is supported by IPA-based analysis in respect to Disease & Function, and also activity-prediction in Functional Networks (Figure 4D). DRPs show a prominent association with protein networks related to Cancer, Organismal Injuries and Abnormalities, and Neurological Diseases (Supplementary Table S5). The neuron-associated network (Figure 4D, network 7 in Supplementary Table S5) highlights the transcription factor TF7L2 as a highly interconnected member. Further, it suggests the downregulation and predicted inhibition of several transcriptional regulators like SOX2, CR3L2, TFE2 and TF7L2 with potential effects on transcription factors of the peroxisome proliferator-activated receptor gamma family (PPARG) (Figure 4D, network 7 in Supplementary Table S5). Indeed, IPA predicted PPARGs as highly significant upstream regulators of diverse DRPs (Supplementary Table S3). Interestingly, proteins of the PPAR family have been reported to modulate inflammatory responses and to be involved in chemotherapy-induced neuropathy (Quintão et al., 2019). In this context, PPARGs have been shown to be regulated by CTNNB1 (β-catenin) (Vallée and Lecarpentier, 2018), a highly upregulated candidate in our pre-CIPN proteome dataset. CTNNB1 acts as a transcriptional co-activator in WNT signaling and is a component of cadherin cell-cell junctions (Wal and Amerongen, 2020). Importantly, we could validate CTNNB1 upregulation at the level of the DRG at pre-CIPN by immunoblotting in an independent cohort of paclitaxel treated rats (Figures 5A,B). IPA functional network analysis predicts CTNNB1 to be connected with prominent signaling pathways such as those of ERK, AMPK, JNK, and alpha catenin (Figure 5C, network 5 in Supplementary Table S5). Several of the candidates in this CTNNB1 network (Figure 5C) were also found to be regulated at pre-CIPN, such as endoplasmin (gene name: *Hsp90b1*; note that, for clarity, in the remainder of the manuscript we additionally indicate gene names, if they differ from protein names entered in the UniProt database, uniprot.org), neural cell adhesion molecule L1 (L1CAM), and collagen alpha-2 (VI) chain (gene name: *Col6A2*) (Supplementary Table S2) suggesting multiple changes within the CTNNB1 network at pre-CIPN. Moreover, the CTNNB1 network harbours proteins previously shown by us to be regulated in another neuropathy-model, i.e., evoked by spared-nerve-injury (SNI) (Rouwette et al., 2016; Barry et al., 2018). Examples (by gene name) include *Hsp90b1*, *Pdia6*, *Aco2*, *Calr*, *Col6A2*, *Tpm4*, and *Vim* just to name a few (Supplementary Table S6).

### Comparison of CIPN-Induced Proteome Dynamics With –Omics Studies on Diverse Neuropathy Models

**FIGURE 6:**
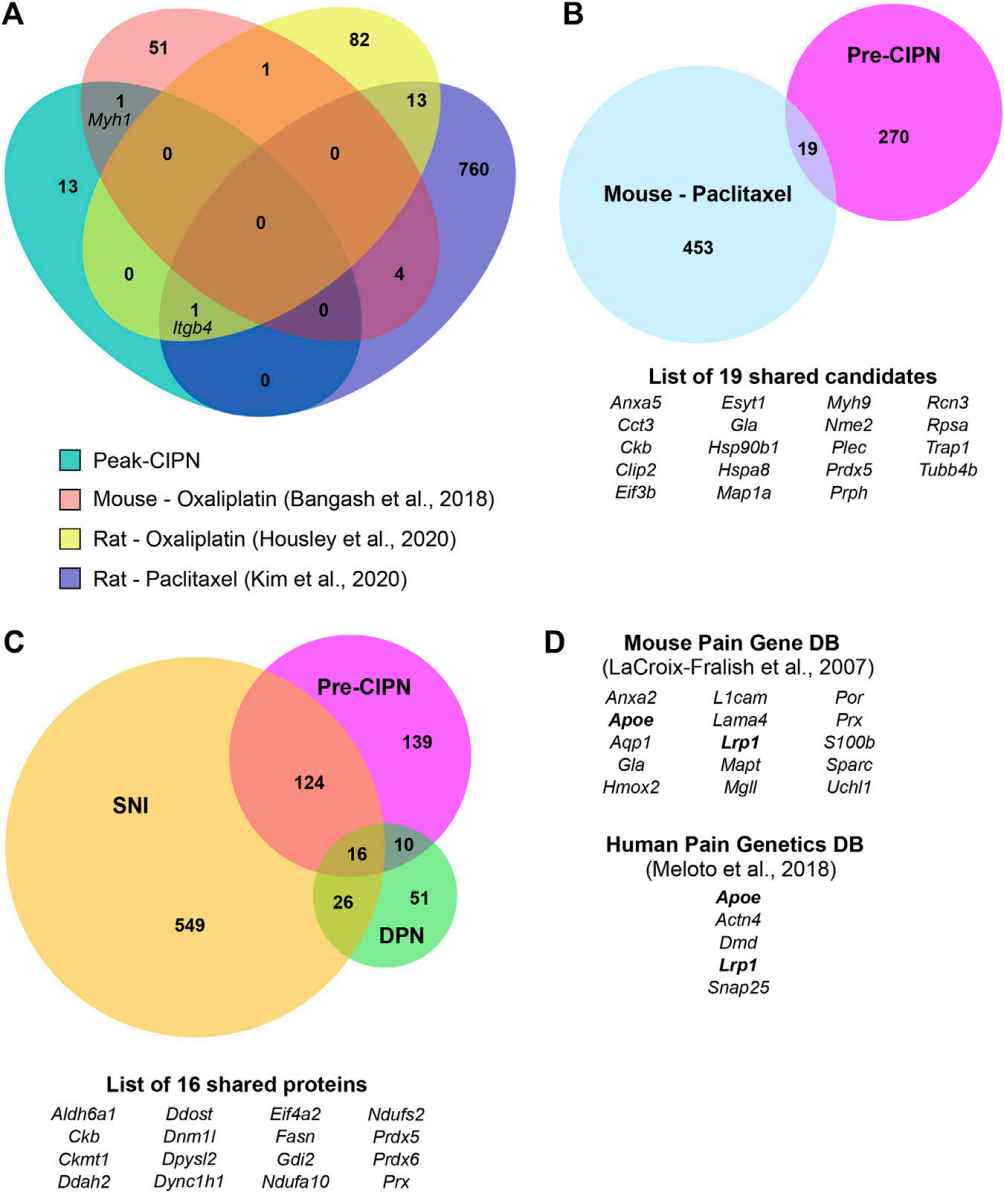
Comparison of our proteome datasets to diverse -omics studies on CIPN and different neuropathy models. **(A)** Venn Diagram for comparison with transcriptome datasets following chemotherapy treatment (Bangash et al., 2018; Housley et al., 2020; Kim et al., 2020) at peak-CIPN. **(B)** Venn Diagram for comparison with the regulated translatome following paclitaxel (Megat et al., 2019) at pre-CIPN. **(C)** Comparison of here reported pre-CIPN-induced proteome alterations with proteome datasets from other chronic pain models: spared nerve inury (SNI) (Barry et al., 2018) and diabetic peripheral neuropathy (DPN) (Akude et al., 2011). Of note, the comparison was performed by gene name, please see methods for details. **(D**) List of genes corresponding to differentially regulated proteins that have previously been associated with a pain phenotype in mice or humans. Bold font: genes that are found in both databases.

This finding prompted us to compare our differentially regulated proteome data at either pre- or peak-CIPN with previously reported datasets. First, we focused on CIPN evoked by oxaliplatin or paclitaxel treatment in rodents. To this end we used published transcriptome and translatome datasets (details in Supplementary Table S7), as thus far proteome data have not been available. The three transcriptome datasets (Bangash et al., 2018; Kim et al., 2020; Housley et al., 2020) were obtained at peak-CIPN (details in Supplementary Table S7), yet we found little overlap (i) among these and (ii) with our results at peak-CIPN (Figure 6A). These discrepancies may indicate the diversity of rodent models, chemotherapeutic agents, exposure regimes, and species as well as strains used across studies. For example, DRG from some mouse strains appear to be more sensitive to the neurotoxic effects of the chemotherapeutic drug cisplatin compared to rat DRG (Podratz et al., 2016). On the other hand, neurite outgrowth of DRG derived from rats and C57BL/6J mice was reported to be more affected by bortezomib treatment than DRG from other tested mouse strains (Podratz et al., 2016). Hence, it is not surprising that only integrin beta (gene name: *Itgb4*) was shared between our data and two other rat datasets using oxaliplatin (Housley et al., 2020) and paclitaxel respectively (Kim et al., 2020) (details in Supplementary Table S7). The translatome dataset by Megat and others (Megat et al., 2019) was generated at pre-CIPN and shared 19 candidates with our differentially regulated protein list at pre-CIPN (Figure 6B; Supplementary Table S7). Among them were endoplasmin (gene name: *Hsp90b1*), mitochondrial proteins peroxiredoxin 5 (PRDX5), tumor necrosis factor type 1 receptor-associated protein (TRAP1), and the eukaryotic translation initiation factor 3 subunit B (EIF3B). However, the direction of protein abundance alterations was consistent only for 36.84% of these candidates (Supplementary Table S7). Again, discrepancies in sample collection (the translatome was obtained from Na_v_1.8 expressing neurons, whereas our proteome data are generated from acutely isolated whole DRG) may account for variability among studies. Similarly, differences in paclitaxel dosage and time points studied need to be considered given the accumulation and persistence of paclitaxel within DRG (reviewed in (Trecarichi and Flatters, 2019). In particular, using a low dosage paclitaxel administration over multiple sessions, such as in the present study, paclitaxel accumulates in rat DRG at day 7, while its concentration is lowered to 1/8 by day 16 (Xiao et al., 2011). Thus, acutely toxic effects of paclitaxel need to be considered when interpreting our pre-CIPN data. In mice, previous reports showed paclitaxel levels to peak in DRG right after the last injection, slowly decrease over time, and low concentrations still being detectable at day 26 (Wozniak et al., 2016).

Next, we aimed at identifying proteins that are commonly regulated across different pain models. To this end we compared our datasets to previously published proteomes, i.e., to differentially regulated proteins identified in a rat model of diabetic peripheral neuropathy (DPN) (Akude et al., 2011) and to differentially regulated proteins in mice upon spared nerve injury (SNI) (Barry et al., 2018). 139/289 (48.10%) proteins altered at pre-CIPN were also altered upon SNI, while only 26/289 (9.00%) where shared with the DPN model (Figure 6C). In total we identified 16 proteins that were shared between all three pain models. Examples include the cytoskeletal protein such a cytoplasmic dynein 1 heavy chain 1 (gene name: *Dync1h1*), periaxin (gene name: *Prx*), mitochondrial proteins, in particular the two NADH dehydrogenases (gene names: *Ndufa10* and *Ndufs2*), as well as peroxiredoxins 5 and 6 (gene names: *Prdx5 and Prdx6*), and the eukaryotic translation initiation factor 4 subunit a2 (gene name: *Eifa2*). For peak-CIPN, only one protein, AHNAK nucleoprotein (gene name: *Ahnak*) was also identified in the DPN proteome, whereas six proteins, including the cytoskeletal protein spectrin alpha (gene name: *Sptan1*) and its beta chain (gene name: *Sptb*), as well as the neurofilament medium polypeptide (gene name: *Nefm*) and aforementioned integrin beta (gene name: *Itgb4*) were shared with the SNI dataset (Supplementary Table S6).

Along the lines of harnessing previous data mining efforts, we also compared our pre-CIPN dataset of differentially regulated proteins with the Mouse Pain Gene database, containing data on gene knockouts leading to a pain phenotype (LaCroix-Fralish et al., 2007), and the Human Pain Genetics database (Meloto et al., 2018). Indeed, in the Mouse Pain Gene database genes corresponding to 15 of our DRPs are included and 5 DRPs have been associated with a pain phenotype in humans (Figure 6D).

## Discussion

This study identifies a specific protein signature in lumbar rat DRG at two critical timepoints for paclitaxel-induced neuropathy using unbiased and quantitative proteome profiling integrated with detailed network analysis. In this way we generated novel insights into molecular mechanisms associated with the development and maintenance of CIPN. First, the vast majority of changes at the level of the DRG appear to happen before a pain phenotype is evident, as suggested by the large number of differentially regulated proteins 7 days after the first treatment. Second, these proteins, of which many are expressed in peripheral sensory neurons, are implicated in multiple biological pathways in the context of mitochondrial (dys)function, immune signaling, and neuronal transcription. Third, subsequent orthogonal validation on the protein level and in an independent cohort validated the increase of β-catenin (CTNNB1) at pre-CIPN and network pharmacology predicts translationally relevant insights into associated and druggable protein networks. Taken together, we provide a unique framework to extend our understanding of mechanisms underlying CIPN from a protein network point of view. In line with emerging approaches of network medicine our study highlights new avenues for developing improved therapeutic options for CIPN, by identifying potential targets for future prevention and treatment.

Here, we performed unbiased proteome profiling of DRG, which identified a defined set of DRPs at two different time points upon paclitaxel treatment (complete list in Supplementary Table S2). Interestingly, about half of DRPs at pre-CIPN were also previously found to be regulated in a severe neuropathic pain model of spared-nerve-injury in mice (Barry et al., 2018) (SNI; at 28 days post-injury, Figure 6C). This significant overlap in the protein signature may indicate the existence of “general pain players” implicated in various neuropathy-related pain entities.

Whole DRG lysates contain proteins from several cell-types, i.e., neurons, fibroblasts, satellite glia cells, immune cells, and also vascular proteins. Due to this cellular complexity and heterogeneity, we cannot assign the detected changes in protein abundance to specific cell types—an issue common to all -omics approaches not performed on the single-cell level. To address this, in part, we compared our datasets with published resources on the transcriptome composition of DRG sensory neurons derived from latest comprehensive single-cell RNAseq studies (Zeisel et al., 2018). Of note, comparisons between transcriptomes and proteomes often show major discrepancies due to the well-known limited predictability of mRNA abundance for protein levels (Reimegård et al., 2021; Liu et al., 2016)—a fact which requires caution in data interpretation. Even so, our results demonstrate a significant overlap with the transcriptome across sensory neuron subpopulations (Figure 4) amounting to 76.82% of DRP at pre-CIPN being expressed in at least one neuronal subset (Figure 4C). In essence, these comparisons suggest that we have profiled the sensory neuron proteome alongside other DRG cell types. Another technical aspect relates to the pooling of DRG from different levels to obtain sufficient tissue per DIA-MS replicate. While this is common practice in most -omics studies, it is noteworthy that even neurons within one DRG are highly heterogeneous, as they innervate different target organs, and affected and non-affected neurons are intermingled upon induction of chronic pain in rodents (Hu et al., 2016; Berta et al., 2017). Despite the use of two independent algorithms to interrogate DIA-MS data, our results do not comprehensively cover proteins known to be expressed in DRG, e.g. sodium channels Na_v_1.7, Na_v_1.8, and Na_v_1.9 known to be involved in several pain states. The reasons can be manifold, ranging from relatively low expression levels to insufficient solubilization or localization in detergent-resistant microdomains—all factors known to render membrane protein analysis challenging (Griffin and Schnitzer, 2011; Prados-Rosales et al., 2019). Nonetheless, our results represent the first dataset on proteome dynamics upon paclitaxel treatment providing unprecedented insights into induced molecular alterations during the development and maintenance of CIPN.

However, any comparison of our data upon paclitaxel treatment with other CIPN studies and their translation to human patients requires caution. Different chemotherapeutic agents may lead to very diverse effects, based on their mechanism of action. Indeed, a study in mice treated with either vincristine, oxaliplatin or cisplatin, found that only 5 DRG genes were dysregulated by all three drugs on the transcript level (Starobova et al., 2020). Moreover, different routes and dosages of paclitaxel (and generally, any chemotherapeutic agent) have been used in rodent models. Ideally, the dosage and formulation of agents would closely mimic regimens of clinical chemotherapy in human patients, i.e., repeated drug administration in several cycles as performed in this study. Finally, drug formulation is another important factor to be considered. While in the clinic the type of formulation largely depends on the type of cancer [reviewed in (Sharifi-Rad et al., 2021)], a recent study indicates that nanoparticle albumin-bound paclitaxel readily accumulates in DRG, but is also cleared faster than Cremophor EL paclitaxel (such as used in this study) (Klein et al., 2021). Thus the extent of direct neurotoxicity of paclitaxel and proteins involved in its clearance may likely differ in dependence on the pharmacokinetics of each formulation.

Besides, differences among sex/genders need to be considered. While most rat CIPN models use male animals, such as we did in this study, it has been shown that, at peak-CIPN, the reduction in nociceptive thresholds is more pronounced in female compared to male rats (Ferrari et al., 2020). Moreover, also in humans, women are more likely to develop CIPN compared to men, while age and BMI are additional risk factors (Mizrahi et al., 2021). In future studies, it would be interesting to determine proteome dynamics in female rats and at different ages to better model the diversity of CIPN patients.

Within the here used experimental CIPN-model, we have demonstrated the validity of our proteomics workflow by confirming the regulation of β-catenin (Figure 5) and extending this knowledge by detailed protein network analysis. In accordance with our findings, recent work showed that active β-catenin is increased in the nucleus of CGRP+ neurons and satellite glial cells of rats 16 days after paclitaxel-induced neuropathy (Kim et al., 2021). In these animals, mechanical hyperalgesia could be reduced by canonical Wnt/β-catenin blockers, which attenuated the increase in paclitaxel-induced levels of active β-catenin at the level of the DRG (Kim et al., 2021). Similarly, mechanical and heat hyperalgesia, as well as cold allodynia produced by CIPN could be reduced by administration of the β-catenin inhibitor PNU76454 at day 21 following the first paclitaxel administration, which also attenuated increased protein levels of β-catenin in the sciatic nerve (Resham and Sharma, 2019). In preclinical studies, PPARG agonists have been shown to present promising therapeutic targets to counteract elevated β-catenin levels and thus have an anti-inflammatory effect (Vallée and Lecarpentier, 2018). β-catenin has also been implicated in other neuropathic pain models, showing a substantial increase in the DRG following chronic constriction injury (CCI) (Li et al., 2015; Xu et al., 2015). In cultured DRG neurons from CCI rats, the nuclear accumulation of β-catenin leads to increased release of substance P, which can be inhibited by Wnt/β-catenin pathway blockers, as well as non-steroidal anti-inflammatory drugs (NSAIDs). Similarly, the NSAID diclofenac is able to prevent mechanical hyperalgesia and accumulation of β-catenin in the rat DRG following CCI (Li et al., 2015). Overall, these findings suggest that activation of the canonical Wnt/β-catenin pathway in DRG might mediate neurogenic inflammation in peptidergic sensory neurons. Further investigations of the downstream effectors of this pathway will help to elucidate if this is the case in CIPN. Here, IPA functional network analysis (Figure 5C) highlights multiple putative interaction partners of β-catenin and thus, potential targets to prevent CIPN, several of which we also identified as differentially regulated.

Our dataset is further supported by previous studies, as several DRPs at pre-CIPN are altered in chronic pain conditions across species: (1) in humans (shared candidates with Human Pain Genetics DB, Figure 6D) and (2) in other rodent models (Figure 6C proteome comparisons and comprehensive literature search). For example, diabetic peripheral neuropathy (DPN) is thought to share energy metabolism-related pathways with CIPN (Flatters and Bennett, 2006; Trecarichi and Flatters, 2019). In line with our peak-CIPN dataset, mRNA of the actin-binding protein *Actn3* and the ATPase *Atp2a1* were increased 4 weeks after induction of diabetic neuropathy in rat DRG, while contrary to our findings, the expression of the myosins *Mylpf* and *Mhy1* was decreased (Yamazaki et al., 2012). Further, the neurofilament medium polypeptide (gene name: *Nefm*), which we found to be slightly decreased at peak-CIPN, shows the same trend in a model of DPN (Xu et al., 2002). Another protein downregulated at peak-CIPN was spectrin-beta. Interestingly, knockout mice of the corresponding gene (*Sptb*) progressively lose Na^+^ channels leading to axonal injury (Liu et al., 2020). Thus, spectrin-beta might play a protective function for neuronal integrity, which is impaired following paclitaxel treatment. Neuron-supporting Schwann cells have also been implied in the pathophysiology of CIPN (Imai et al., 2017). Integrin beta (gene name: *Itgb4*), which was downregulated in our dataset and two other CIPN transcriptomes at peak-CIPN in rats, has been implicated in neuronal regeneration mediated by Schwann cells (Zee et al., 2008). Moreover, AHNAK, our top downregulated protein at peak-CIPN, is expressed in Schwann cells, where it mediates myelin maintenance (Salim et al., 2009). Thus, while we only identified few altered proteins at peak-CIPN, they point towards an important role of neuronal maintenance/support at advanced stages of the pathology.

Neuronal damage seems to play a role already before pain develops, based on the proteins we found to be dysregulated at pre-CIPN. For example, extended synaptogamin-1 (ESYT1), implicated in the replenishment of ER Ca^2+^ stores (Chang et al., 2013), has been found to be upregulated in a mouse model of CIPN (Megat et al., 2019). Complement 3 (C3), which we found to be prominently upregulated at pre-CIPN, has been shown to be induced by paclitaxel *in vitro* leading to neuronal damage (Xu et al., 2018). Remarkably, C3 knockout rats show reduced damage to dermal fibers upon CIPN, providing a viable target for therapeutic intervention (Xu et al., 2018). Advillin (AVIL), which was upregulated in our dataset at pre-CIPN, might play a neuroprotective role and deletion of the gene impairs neuronal regeneration specifically in IB4+ neurons (Chuang et al., 2018), a cell population which we have previously implicated in the pathophysiology of CIPN (Duggett et al., 2016). Additionally, *Advillin* knockout mice develop more severe cold allodynia in response to oxaliplatin treatment than littermates (Chuang et al., 2018). Overall, our findings suggest that several changes occur at the level of the DRG, particularly in neuronal proteins, prior to pain development. On the other hand, at later stages – when neuropathy has fully developed – proteins implicated in supporting neuronal health/regeneration (e.g., those expressed in Schwann cells) seem to play a prominent role.

In conclusion, we identified, in an unbiased and quantitative manner, differentially regulated proteins in DRG after *in vivo* paclitaxel exposure, prior to neuropathy development and, separately, when paclitaxel-induced neuropathy was fully established. *Via* orthogonal validation, data mining, and extensive network analysis we provide evidence for the high utility of our dataset: Our results may explain the underlying mechanisms following *in vivo* paclitaxel and its neurotoxic side-effects by providing unprecedented insights into the dynamics of induced molecular changes and biological pathways. These findings represent a unique source for future work on the functional impact of manipulating here identified proteins and, in particular, associated protein networks. Taken together, our study provides a stepping stone for translational research aimed at developing novel therapeutics for the prevention and treatment of CIPN in line with emerging network medicine.

## Data Availability

The datasets presented in this study can be found in online repositories. The names of the repository/repositories and accession number(s) can be found below: ProteomeXchange Consortium via the PRIDE partner repository; PXD028356.
