# Supplementary figures and images for: Proteome and Network Analysis Provides Novel Insights Into Developing and Established Chemotherapy-Induced Peripheral Neuropathy
Source: Front Pharmacol. 2022 Feb 18;13:818690. doi: 10.3389/fphar.2022.818690 (PMC8895144; doi:10.3389/fphar.2022.818690)

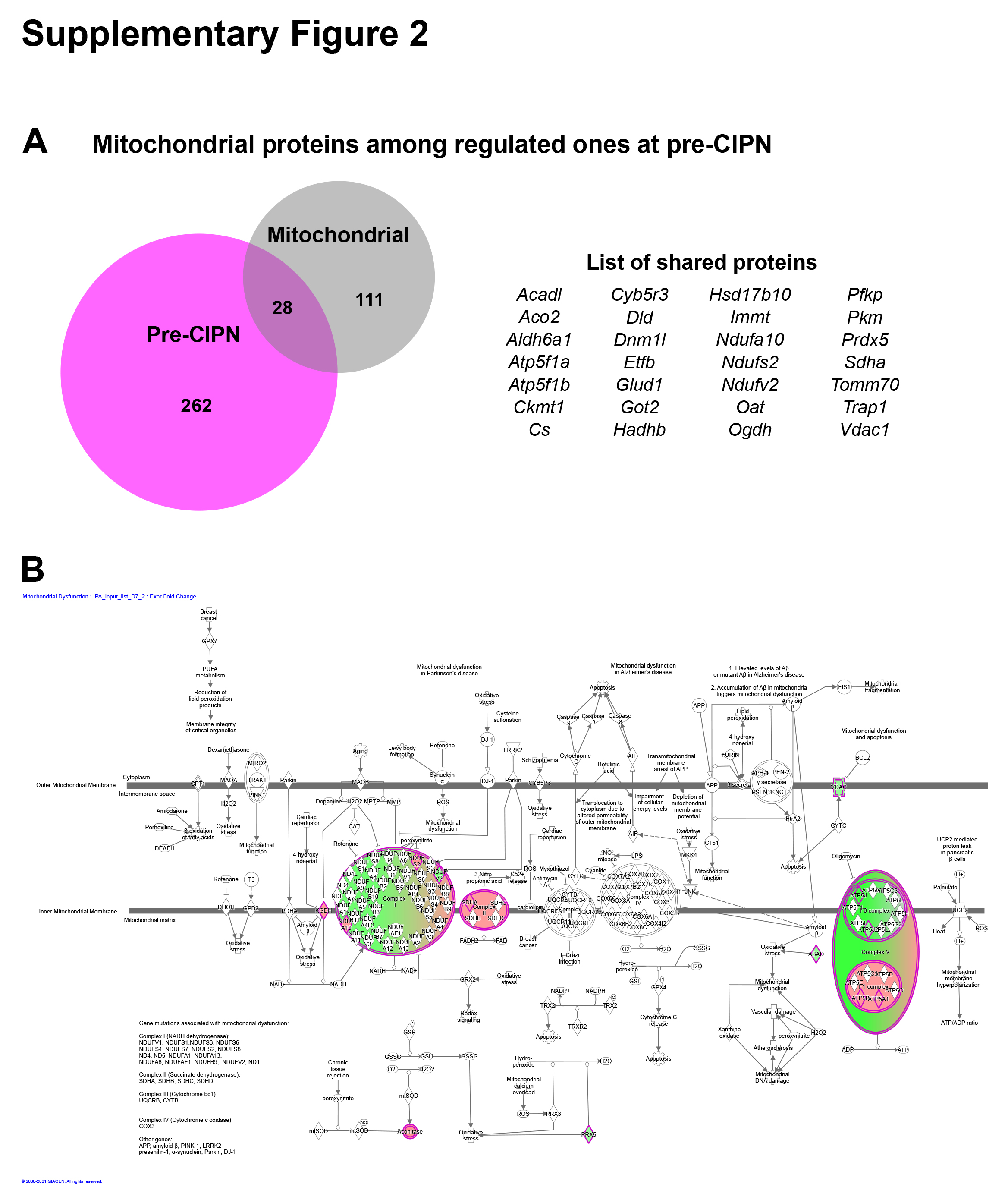

Supplement: Supplementary file 3 [file Image2.jpg]

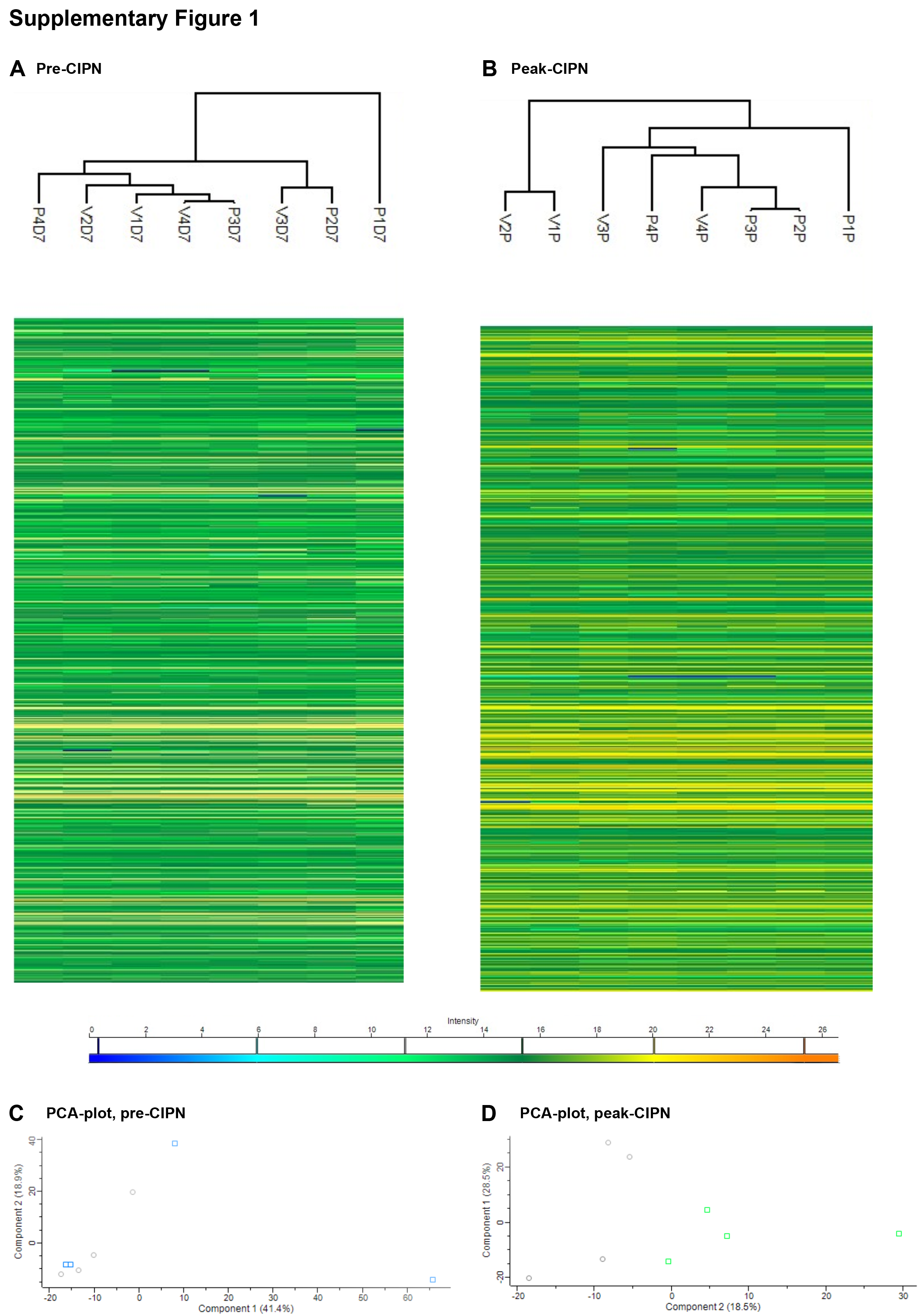

Supplement: Supplementary file 4 [file Image1.TIF]
