# Supplementary material for: Proteome and Network Analysis Provides Novel Insights Into Developing and Established Chemotherapy-Induced Peripheral Neuropathy
Source: Front Pharmacol. 2022 Feb 18;13:818690. doi: 10.3389/fphar.2022.818690 (PMC8895144; doi:10.3389/fphar.2022.818690)
Supplement: Supplementary file 10 [file DataSheet1.docx]

Supplementary Material

**Supplemental Figure 1** (A-B) Heatmap of unsupervised clustering of z-scored
normalized protein intensities obtained by DIA-MS (data from spectral library-based analysis pipeline, please see Methods for details) from all analyzed biological replicates at pre-CIPN (A) and peak-CIPN (B). The legend color bar indicates the range of z-scores within rows. (C-D) Principal component analysis (PCA) of all analyzed biological replicates at pre-CIPN (C) and peak-CIPN (D). Squares: biological replicates from paclitaxel-treated rats; circles: biological replicates from vehicle-treated rats.

**Supplemental Figure 2** (A) Venn diagram illustrating the overlap of pre-CIPN proteins with the DRG mitochondrial rat proteome and list of all shared proteins. (B) Schematic representation of protein networks associated with mitochondrial dysfunction as obtained by QIAGEN Ingenuity Pathway Analysis (IPA). Mean protein abundance changes of significantly regulated proteins across all replicates at pre-CIPN are coded by a color gradient. Color code: red, upregulated; green, downregulated; grey, unchanged; white, not detected.

**Supplemental Table 1** Complete dataset of comparative proteomics results at pre-CIPN and peak-CIPN. Only proteins identified with two or more unique peptides (column M) are considered.

**Supplemental Table 2** Candidate list of all protein groups that are dysregulated at pre- and peak-CIPN. Benjamini-Hochberg (BH)-adjusted p-values (i.e. q-values) were used for multiple testing and differentially regulated proteins (DRPs) were defined by setting a cutoff of q < 0.05. Single-peptide hits (unique peptides < 2) and potential keratin and serum albumin contaminations were removed. Six protein groups highlighted in yellow were excluded from comparison with other datasets (performed on the level of gene names given the nature of most other datasets) as no unique gene name has been assigned.

**Supplemental Table 3** QIAGEN Ingenuity Pathway Analysis (IPA) of DRPs at pre-CIPN for canonical pathways, toxicity list, diseases and biological function, and upstream regulators. 4/295 DRPs could not be mapped due to missing information in the IPA database.

**Supplemental Table 4** Comparison of DRPs at pre- and peak-CIPN with basal expression of corresponding genes in neuronal subsets based on mouse single cell RNAseq data [21].

**Supplemental Table 5** Protein networks predicted to be associated with DRPs at pre-CIPN as identified by IPA.

**Supplemental Table 6** Comparison of DRPs at pre- and peak-CIPN with other published proteomes for the neuropathic pain models SNI [41] and DPN [43], and the mitochondrial proteome [43].

**Supplemental Table 7** Comparison of DRPs at pre- and peak-CIPN with published transcriptomes (differentially expressed genes, DEG) [7–9] and translatome datasets [6] in other models of CIPN.
